# Investigating protein‐membrane interactions using native reverse micelles constructed from naturally sourced lipids

**DOI:** 10.1002/pro.4786

**Published:** 2023-10-16

**Authors:** Sara H. Walters, Abdul J. Castillo, Angela M. Develin, Courtney L. Labrecque, Yun Qu, Brian Fuglestad

**Affiliations:** ^1^ Department of Chemistry Virginia Commonwealth University Richmond Virginia USA; ^2^ Institute for Structural Biology, Drug Discovery and Development Virginia Commonwealth University Richmond Virginia USA

**Keywords:** fatty acid binding protein 4, glutathione peroxidase 4, membrane models, peripheral membrane proteins, phosphatidylethanolamine‐binding protein 1, protein NMR, protein‐membrane interactions, reverse micelles, ubiquitin

## Abstract

Advancing the study of membrane associated proteins and their interactions is dependent on accurate membrane models. While a variety of membrane models for high‐resolution membrane protein study exist, most do not reflect the diversity of lipids found within biological membranes. In this work, we have developed native reverse micelles (nRMs) formulated with lipids from multiple eukaryotic sources, which encapsulate proteins and enable them to interact as they would with a biological membrane. Diverse formulations of nRMs using soy lecithin, porcine brain lipids, or bovine heart lipids combined with *n‐*dodecylphosphocholine were developed and characterized by dynamic light scattering and ^31^P‐NMR. To optimize protein encapsulation, ubiquitin was used as a standard and protein NMR verified minimal changes to its structure. Peripheral membrane proteins, which bind reversibly to membranes, were encapsulated and include glutathione peroxidase 4 (GPx4), phosphatidylethanolamine‐binding protein 1 (PEBP1), and fatty acid binding protein 4 (FABP4). All three proteins showed anticipated interactions with the membrane‐like inner surface of the nRMs as assessed by protein NMR. The nRM formulations developed here allow for efficient, high‐resolution study of membrane interacting proteins up to and beyond ~21 kDa, in a more biologically relevant context compared to other non‐native membrane models. The approach outlined here may be applied to a wide range of lipid extracts, allowing study of a variety of membrane associated proteins in their specific biological context.

## 1 INTRODUCTION

The interplay between proteins and membranes is complex and driven by factors such as the amino acid sequence of the protein and the identity of the lipids within the membrane. A wide variety of membrane‐interacting proteins exist, including integral membrane proteins, which span the lipid bilayer, membrane anchored proteins, which are attached to the membrane via covalent lipidation or amphipathic structural elements, and peripheral membrane proteins (PMPs), which are water‐soluble proteins that interact reversibly with membranes (Chou & Elrod, 1999; Whited & Johs, 2015). The significance of interactions between membranes and proteins in disease and basic biological function gives rise to a great demand to investigate and understand this class of proteins. Experimental high‐resolution observation of protein‐membrane and protein‐lipid interactions is challenging due to various technical barriers (Carpenter et al., 2008; Cheng, 2018). While strides have been made in a variety of methods, such as cryo‐EM and solid‐state NMR, a high‐resolution views of many crucial interactions between proteins with membranes and lipids remain elusive (Cheng, 2018; Lacapere et al., 2007). Solution‐state NMR is well‐suited for illuminating protein interactions with membranes and lipids, however current membrane models have limitations. Commonly used membrane models for NMR structural and functional studies include micelles, bicelles, and nanodiscs, each with their own advantages and disadvantages (Frey et al., 2017; Sanders & Sönnichsen, 2006; Warschawski et al., 2011). While nanodiscs and bicelles are excellent tools for protein NMR, they are relatively large assemblies that often require deuteration for even modestly sized proteins (Puthenveetil et al., 2017; Yeh et al., 2020). Micelles have long been used as membrane models in protein NMR. However, they are most often constructed from highly artificial detergents, which often affects stability and structure of membrane associated proteins in these systems (Chipot et al., 2018; Seddon et al., 2004).

**FIGURE 1:**
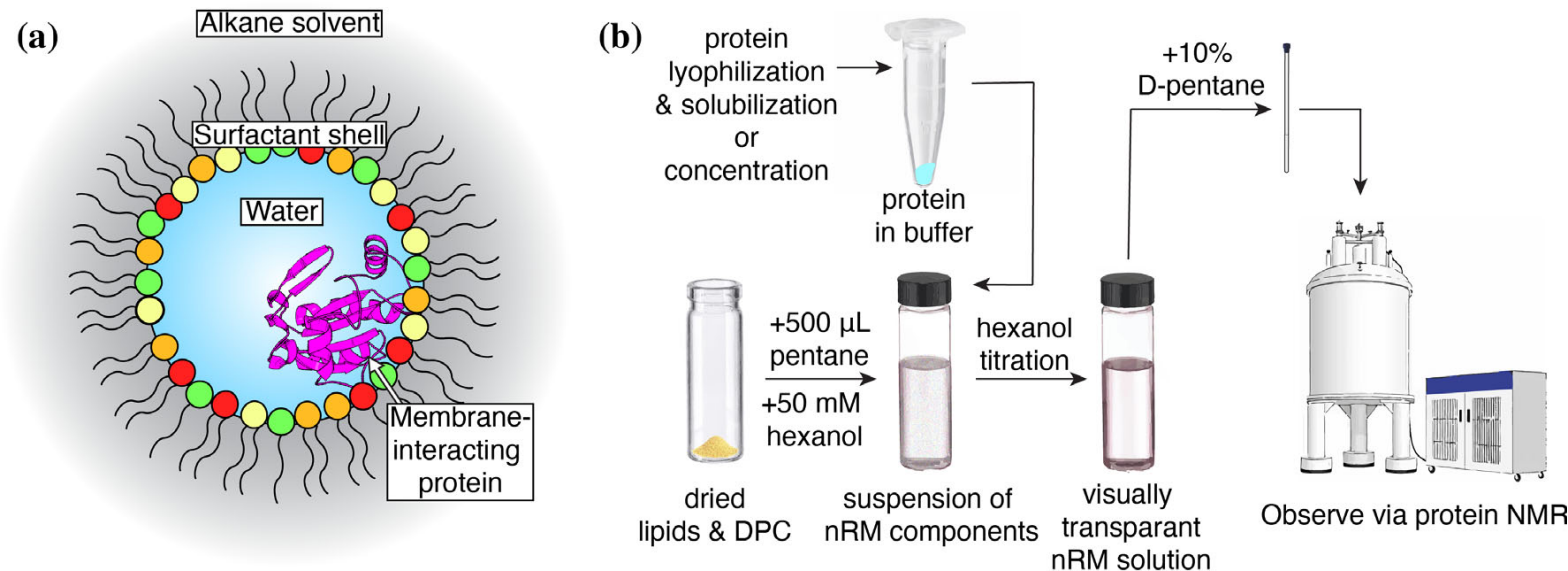
(a) Cartoon of a peripheral membrane protein (PMP) encapsulated within a native reverse micelle (nRM) and binding to the interior surface. (b) General scheme of protein encapsulation within nRMs for NMR experiments. Vigorous vortex mixing is performed upon addition of solvent, addition of protein solution, and between every step of the hexanol titration. After the sample is constructed, often determined by visual clarity, 10% d‐pentane is added as the NMR lock solvent.

Use of reverse micelles (RMs) as membrane models has been demonstrated for membrane‐anchored and integral membrane proteins (Kielec et al., 2009; Valentine et al., 2010). RMs are approximately spherical shells of surfactants that surround a nano‐pool of water or other polar solvent, solubilized in a bulk alkane (Figure 1a) (Chevalier & Zemb, 1990; Correa et al., 2012; Pileni, 1989). Proteins may be housed within an aqueous interior of RMs (Luisi, 1985; Luisi et al., 1988; Wand et al., 1998). RM encapsulation combined with protein NMR enables a variety of unique protein studies including forced folding from confinement (Peterson et al., 2004), hydration dynamics measurements (Nucci et al., 2011), experimental cosolvent mapping (Fuglestad, Kerstetter, & Wand, 2019), and enhanced fragment screening for inhibitor design (Fuglestad, Kerstetter, Bédard, & Wand, 2019). RM development for protein applications has generally focused on aqueous proteins with minimal perturbations to structure upon encapsulation as a major goal (Dodevski et al., 2014; Nucci et al., 2014). A recent advance used phosphatidylcholine surfactants to develop membrane‐mimicking reverse micelles (mmRMs) constructed from a mixture of 1,2‐dilauroyl‐sn‐glycero‐3‐phosphocholine (DLPC) and *n‐*dodecylphosphocholine (DPC) with a 1‐hexanol cosurfactant (Labrecque et al., 2022). The resulting assembly in‐part models cellular membranes and encapsulates PMPs in their membrane‐embedded state. Though the mmRM is a relatively large assembly, the alkane solvent used to solubilize mmRMs is typically pentane or hexane. These low viscosity solvents result in faster tumbling and more favorable NMR spectroscopic conditions for proteins up to ~25 kDa without the need for protein deuteration or TROSY methods (Pervushin et al., 1997). Shorter‐chain, lower viscosity alkanes such as propane and ethane enable even larger proteins to be studied without the need for perdeuteration (Fuglestad, Marques, Jorge, et al., 2019; Peterson et al., 2005). Additionally, proteins are highly stable when encapsulated within the mmRMs, with lifetimes exceeding months (Labrecque et al., 2022). Development of mmRMs has expanded the available membrane model systems for efficient study of membrane associated proteins.

Membrane models, including mmRMs, are most often constructed from artificial detergents or are highly homogeneous in their composition, typically containing one or two types of lipids or detergents. While generally useful for high‐resolution study of membrane related proteins, they deviate significantly from natural membranes which contain a tremendous chemical diversity of lipids (Harayama & Riezman, 2018). The presence of a milieu of lipids within cellular membranes likely influences the binding preference, structure, dynamics, and other key aspects of protein function (Levental & Lyman, 2023; Palsdottir & Hunte, 2004; Whited & Johs, 2015). Measurements conducted within a natural lipid mixture would most accurately capture these protein properties. Natively sourced lipids have been used to construct bicelles and nanodiscs, which have provided tools that are used in conjunction with NMR and other methods to study proteins in a more natural environment (Brown et al., 2021; Smrt et al., 2017).

Here we present the use of native reverse micelles (nRMs) as membrane models for the high‐resolution study of PMPs. Inspired by previous formulations of RMs using soybean lecithin (SL) (Walde et al., 1990; Willard et al., 1998), we have applied this natural phospholipid extraction to construct nRMs that mimic the lipid content of natural membranes. We extend on this to utilize lipids extracted from animal sources, including bovine heart lipids (BHLs) and porcine brain lipids (PBLs), for construction of nRMs. We encapsulated a total of four proteins within all three nRM systems. Ubiquitin is a commonly used NMR standard and is used here as a model for water‐solubilized proteins. Three human PMPs are encapsulated to characterize the interactions of the protein with the membrane‐like inner surface of nRMs; glutathione peroxidase 4 (GPx4), phosphatidylethanolamine‐binding protein 1 (PEBP1), and fatty acid binding protein 4 (FABP4). GPx4 is a central regulator of ferroptosis, which is cell death by over‐hydroperoxidation of lipids, and functions by reducing lipid hydroperoxides (Cozza et al., 2017; Forcina & Dixon, 2019). It has emerged as an important drug target, with one major goal being to leverage ferroptosis to kill therapy resistant cancer cells via targeted inhibition of GPx4 (Hangauer et al., 2017). PEBP1, or raf‐1 kinase inhibitory protein, is responsible for modulating multiple signaling pathways through protein–protein interactions and membrane engagement (Rajkumar et al., 2016; Schoentgen & Jonic, 2020). FABP4 is a lipid chaperone that shuttles fatty acids among membranes and is a drug target implicated in a variety of metabolic related diseases, such as diabetes and metabolic disorders (Floresta et al., 2017; Prentice et al., 2019). The three PMPs investigated here are expected to interact with a single membrane leaflet, driven by electrostatic interactions with the membrane surface and possibly hydrophobic sidechains partitioning towards the nonpolar membrane interior (Whited & Johs, 2015). For all three of the nRM formulations, all proteins were successfully encapsulated, and NMR spectra were acquired, demonstrating robustness of the approach. The nRM protein encapsulation method presented here provides a useful tool that may be used to measure a wide variety of protein‐membrane and protein‐lipid interactions at high‐resolution within a more biologically‐accurate context.

## 2 RESULTS

Successful protein encapsulation within RMs requires three major factors: a surfactant mixture, protein in an aqueous buffer, and a low viscosity solvent such as pentane, which reduces tumbling times for improved NMR performance. Optimized mixtures of these components readily form RMs under conditions with limited volumes of the aqueous phase (Luisi et al., 1988). The initial surfactant selected here was purified natural SL. Initial formulations were screened using visual clarity as a metric for potential RM formation (Figure S1A) (Fuglestad, Marques, Jorge, et al., 2019; Labrecque et al., 2022). We found that soy lecithin at 2.42–3.38 m/v%, with 22.5–37.5 mM DPC, 200–400 mM hexanol, and a water loading (*W*_0_, molar ratio of water to DPC and lecithin) of 10–30 formed RMs, as evidenced by fully transparent solutions (Figures 1b and S1A). We note that the lipid extractions are mixtures of lipids of varying molecular weight, making m/v% an appropriate measure of material used. However, analysis of ^31^P spectra and integration allows us to approximate a mixture containing 2.42 m/v% soy lecithin with 37.5 mM DPC as a 50:50 molar percent ratio, and a mixture of 3.38 m/v% soy lecithin with 22.5 mM DPC as a 70:30 ratio. For convenience, we will use these approximate molar ratios through the manuscript to describe mixtures of DPC with the above mass to volume ratios of lipid extracts. The clarified nRM solutions were initially observed and characterized using ^1^H‐decoupled, ^31^P 1D NMR to estimate the phospholipid content of each lipid mixture and molar ratio of DPC to lipid extract. However, these experiments revealed severe line‐broadening that we attribute to paramagnetic metal contamination of the commercially purified soy lecithin (Meneses & Glonek, 1988). Analysis of lipid content using ^31^P NMR spectra was not possible under these conditions due to the severe line‐broadening. Using an EDTA chelation and phase extraction method, we were able to remove metals and observe narrow line shapes in the ^31^P spectrum (Figure S2A). Removal of the metals using the EDTA treatment has only a minor effect on nRM size (Figure S2B). We sought to avoid this potential source of protein signal degradation and used EDTA treated SL from this point forward. The greatly improved ^31^P spectra allowed us to determine the lipid types that are present, categorized by headgroup, and percent composition (Figure S2C). Assignments of soy lecithin ^31^P 1D‐NMR spectra of the nRMs were determined by those previously published and confirmed by comparing to known lipids added to the nRM system (Glonek, 1998; Kato et al., 2018). Compositional analysis of phospholipids according to headgroup type is summarized in Table S1. In brief, SL contains mostly phosphatidylcholine (PC, ~38%), phosphatidylethanolamine (PE, ~32%), and phosphatidylinositol (PI, ~19%) type lipids, with small percentages of other phospholipid types.

We then expanded our screening to include PBL and BHL polar extracts. 50:50 mixtures of BHL:DPC and PBL:DPC were found to form clarified solutions with 0.60–1.1 M hexanol and *W*_0_ values ranging from 10 to 30. The percent composition reported by the supplier (Avanti Polar Lipids) for PBL were confirmed by ^31^P 1D NMR using a 50:50 PBL:DPC empty nRM (Table S1, Figure S2D), which was also used to estimate the PBL:DPC molar ratio. In addition to large percentages of PC (~13%), PE (~33%), and phosphatidylserine (PS, ~19%), other lipid types such as PI and phosphatidic acid (PA) are present in smaller proportions. A fraction (~31%) of the lipid content is reported as unknown by the supplier. Based on known tissue distributions, approximately 20% of the total composition is cholesterol, which is not visible on the ^31^P 1D NMR, with the remaining unknown lipid types anticipated to be composed of sterols, sphingolipids, and acylglycerides (Lai et al., 2016). The BHL lipid compositions reported by the supplier (Avanti Polar Lipids) were verified by ^31^P 1D NMR using 50:50 BHL:DPC nRMs (Table S1, Figure S2E), again used to estimate and confirm the BHL:DPC molar ratio (Metz & Dunphy, 1996). In addition to the reported phospholipids, mostly PE (~14%) and PC (~8.6%), ~58% of the mass of BHL is reported as neutral lipids. Tissue lipid distribution suggests that ~44% of the total lipids are triacylglycerides and ~8% is cholesterol, accounting for most of the unreported neutral lipid fraction (Das & Rouser, 1967). Other sterols, other acylglycerides, and sphingolipids are expected to compose the remaining neutral lipid fraction and the ~17% unknown reported by the supplier. The three lipid extracts used here have a broad range of lipid contents with diverse lipid types, making them useful mixtures for establishing nRMs of varying lipid content.

**FIGURE 2:**
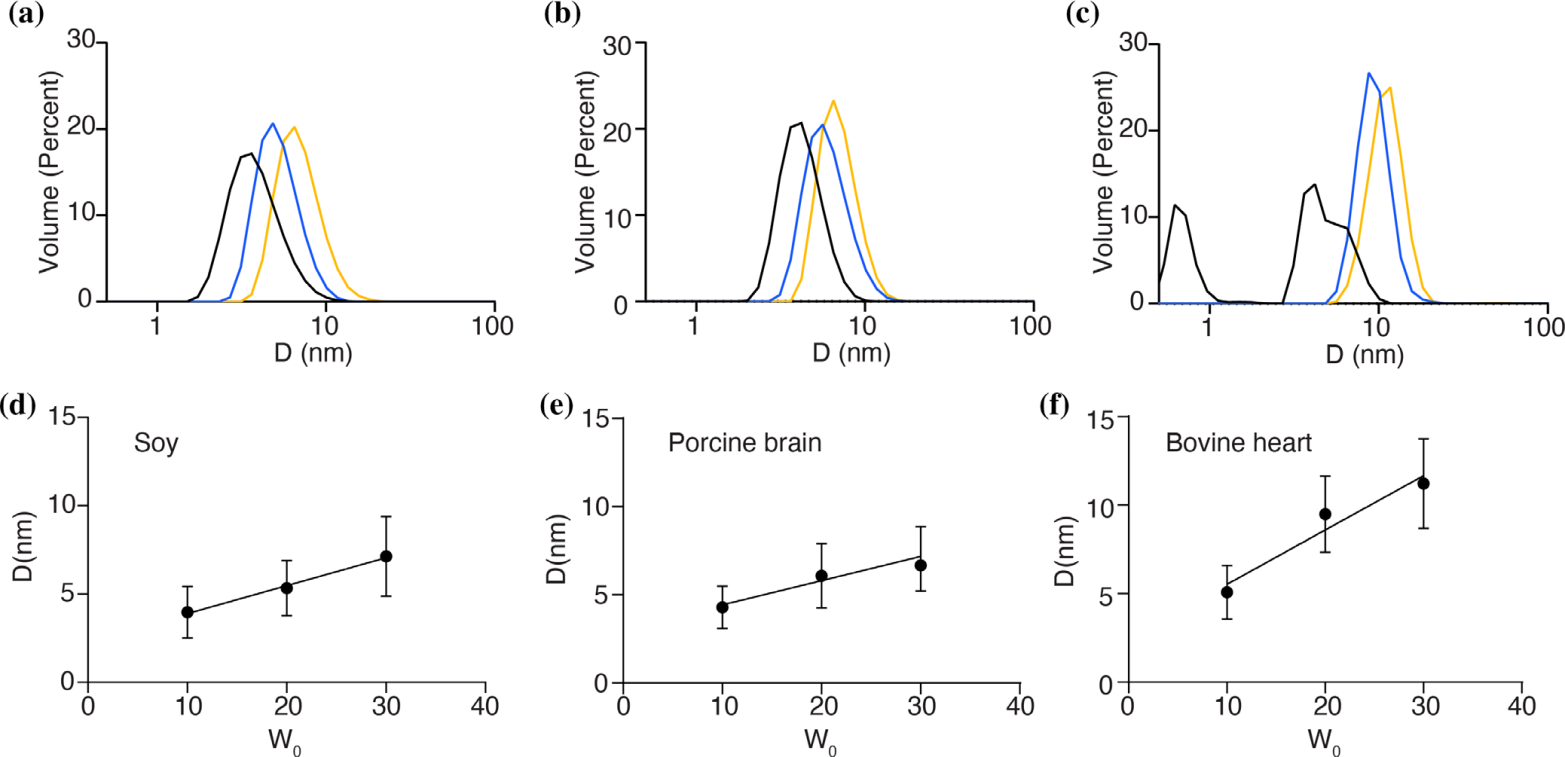
Native reverse micelle (nRM) solvodynamic diameter increases as the water loading increases with natural lipid mixtures. (a) DLS distributions for *W*_0_ of 10 (black), 20 (blue), and 30 (orange) for 50:50 soy lecithin (SL):*n‐*dodecylphosphocholine (DPC). (b) DLS distributions for *W*_0_ of 10 (black), 20 (blue), and 30 (orange) for 50:50 porcine brain lipid (PBL):DPC. (c) DLS distributions for *W*_0_ of 10 (black), 20 (blue), and 30 (orange) for 50:50 bovine heart lipid (BHL):DPC. (d) Average diameters of empty nRMs formulated from 75 mM mixture of 50:50 SL:DPC after heavy metal chelation at *W*_0_ = 10 (4.0 ± 1.5 nm), 20 (5.3 ± 1.6 nm), and 30 (7.1 ± 2.2 nm). (e) Average diameters of empty nRM formulated from 75 mM mixture of 50:50 PBL:DPC at *W*_0_ = 10 (4.3 ± 1.2 nm), 20 (6.1 ± 1.8 nm), and 30 (7.0 ± 1.8 nm). (f) Average diameters of empty nRM formulated from 75 mM mixture of 50:50 BHL:DPC at *W*_0_ = 10 (5.1 ± 1.5 nm, the larger of the two distributions), 20 (9.5 ± 2.2 nm), and 30 (11.2 ± 2.5 nm). Error bars represent the standard deviation of the size distribution determined by dynamic light scattering.

Before proteins were introduced, we pursued dynamic light scattering (DLS) measurements to understand the size and shape characteristics of the nRMs and confirm RM formation. DLS analysis of all three lipid mixtures showed small, monodisperse, and approximately spherical assemblies under the conditions tested, confirming formation of RMs (Figure 2a–c), with the exception of BHL:DPC nRMs at *W*_0_ = 10, which formed a biphasic size distribution. At *W*_0_ = 20 and 30, the distribution of BHL:DPC nRMs were monodisperse and reflected small, spherical RMs (Figure 2c). Upon addition of water, the approximate size of the nRMs increased, as expected for RMs (De & Maitra, 1995; Fuglestad, Gupta, Wand, & Sharp, 2019; Mills et al., 2014; Palazzo et al., 2003). For a 50:50 SL:DPC mixture, the solvodynamic diameter determined by DLS increased from 4.0 ± 1.6 nm to 7.1 ± 2.2 nm with the addition of water from *W*_0_ = 10 to *W*_0_ = 30 (Figure 2d). Here we report the RM size with the standard deviation of the size distribution from DLS measurements. For 70:30 SL:DPC nRMs the solvodynamic diameter determined by DLS increased from 4.2 ± 1.3 nm at *W*_0_ = 10 to 6.0 ± 1.6 nm at *W*_0_ = 30 (Figure S3). The PBL:DPC nRMs showed a similar linear trend with increasing water loading, increasing the solvodynamic diameter from 4.3 ± 1.2 nm at *W*_0_ = 10 to 7.0 ± 1.8 nm at *W*_0_ = 30 (Figure 2e). The BHL solvodynamic diameter, as determined by DLS, increased from 5.1 ± 1.5 nm (for the larger of the two species) to 11.2 ± 2.5 nm as *W*_0_ was increased from 10 to 30 (Figure 2f). In addition to the biphasic behavior observed at *W*_0_ = 10, BHL:DPC nRMs are somewhat larger compared to SL:DPC and PBL:DPC nRMs.

**FIGURE 3:**
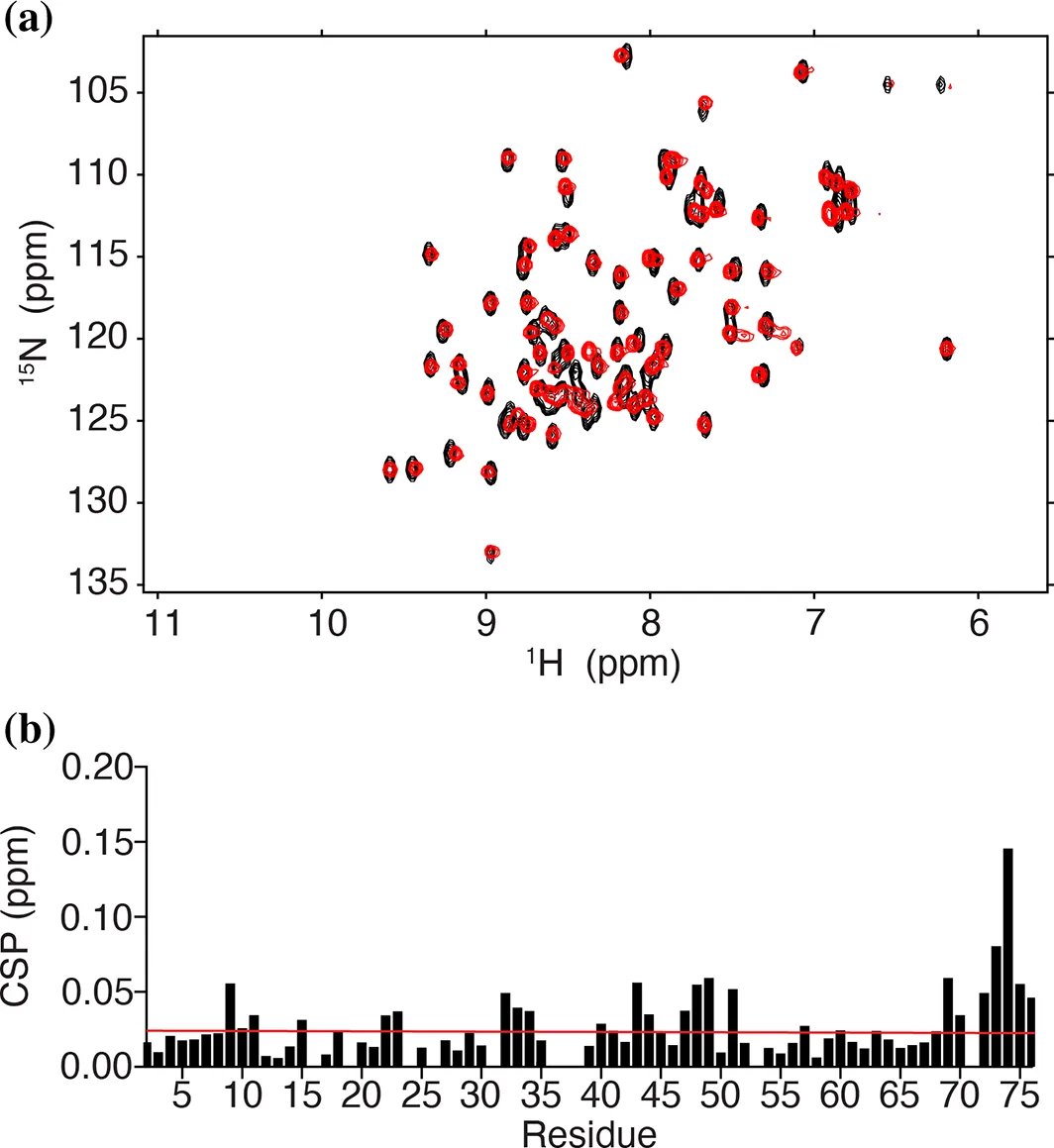
Optimal conditions of native reverse micelle (nRM) formulation are determined with ubiquitin encapsulation. (a) ^15^N‐HSQC of aqueous ubiquitin pH 6.0 (black) and encapsulated ubiquitin (red) in 75 mM 50:50 soy lecithin (SL):*n‐*dodecylphosphocholine (DPC) nRM at a *W*_0_ of 20 and 500 mM hexanol. (b) Encapsulated ubiquitin shows anticipated shifting of surface residues due to weak anionic interactions. Interactions were quantified by calculating the 20% trimmed mean and adding 1σ (0.022 ppm, red line).

To optimize protein encapsulation, we first tested ubiquitin within SL:DPC nRMs with varying W_0_ values and hexanol concentrations. We collected ^1^H‐^15^N HSQC spectra to determine structural fidelity to the aqueous state and were able to unambiguously map 91% of all residues to their corresponding resonances. Initial encapsulation with *W*_0_ = 10 resulted in a high‐quality spectrum, but a series of residues displayed relatively large chemical shift differences compared to the aqueous spectrum (Figure S4A). Increasing *W*_0_ to 15 moves resonances closer to the aqueous spectrum chemical shifts, with a *W*_0_ = 20 matching very closely with the aqueous spectrum (Figures 3a and S4A). Based on comparison of encapsulated ubiquitin to an aqueous pH titration, we were able to determine that SL nRMs shift the ubiquitin aqueous phase from a pH of 5.5 to 6.0 when encapsulated, indicating that the lipid headgroups affect the pH of the system to a small degree (Figure S4B, C). Here, care must be taken to use only pH sensitive resonances that do not have shifting due to interaction with the nRM. Even after accounting for the pH change, some resonances for nRM encapsulated ubiquitin display significant chemical shift perturbations (CSPs) compared to the aqueous spectrum at pH 6.0. Residues T9, T22, D32, L43, K48, Q49, E51, L69, and R72‐G76 all shift more than a 20% trimmed mean (truncating the highest 20% CSPs only) plus 1σ (0.022 ppm, Figure 3b). Ubiquitin is known to interact weakly with anionic surfaces and these residues match well with those that are known to form the interface (Labrecque et al., 2022; Nucci et al., 2011; Shaw et al., 2011). Throughout these tests, 1‐hexanol was titrated into the samples, from an initial concentration of 50 mM, until full sample clarity was achieved. Optimal 1‐hexanol concentrations were found to be ≥500 mM. The result suggests that a minimum of *W*_0_ = 20 and ≥500 mM 1‐hexanol is optimal for protein encapsulation in the SL:DPC nRM system. Using a 50:50 PBL:DPC ratio, ubiquitin successfully encapsulated at a *W*_0_ = 20 (Figure S5A). The 50:50 BHL:DPC mixture also successfully encapsulated ubiquitin at *W*_0_ = 20 (Figure S5B). The encapsulations were verified by ^15^N‐HSQC. Based on the aqueous pH titration, there was a small shift to pH 6.0 when ubiquitin was encapsulated with PBL and BHL and chemical shifts were compared against the pH 6.0 aqueous spectrum (Figure S4D, E). Encapsulation in PBL and BHL based nRMs show modest shifting of ubiquitin spectral resonances (Figure S5C,D), comparable to shifting observed in SL:DPC nRMs with *W*_0_ = 20 (Figure 3b). This effect was not previously observed for DLPC:DPC mmRMs (Labrecque et al., 2022).

**FIGURE 4:**
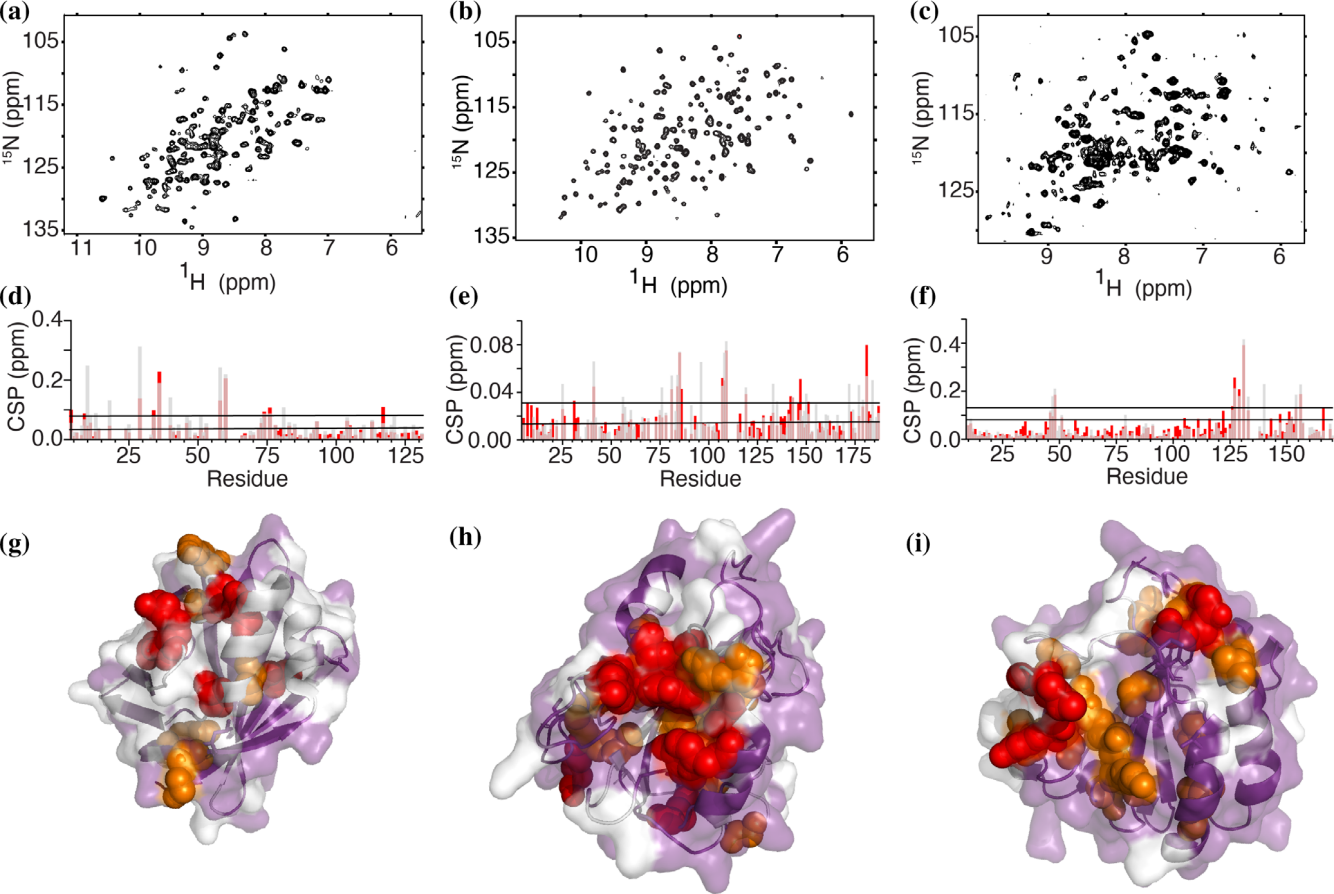
Encapsulation of FABP4, PEBP1, and GPx4 in soy lecithin (SL):*n‐*dodecylphosphocholine (DPC) native reverse micelles (nRMs). (a) ^15^N‐HSQC of FABP4 encapsulated in 75 mM of 70:30 SL:DPC nRM at a *W*_0_ of 25 with 300 mM hexanol. (b) ^15^N‐HSQC of PEBP1 encapsulated in 75 mM of 50:50 SL:DPC nRM at a *W*_0_ of 25 with 800 mM hexanol. (c) ^15^N‐HSQC of GPx4 encapsulated in 75 mM of 70:30 SL:DPC nRM at a *W*_0_ of 25 with 400 mM hexanol. (d) Chemical shift perturbation (CSP) comparison of 50:50 DLPC:DPC (gray) and 70:30 SL:DPC (pH 6.5; red) of FABP4 show small differences between the two encapsulations. Significantly shifting resonances were determined by calculating a 20% trimmed mean plus 1σ (0.04 ppm) and 2σ (0.08 ppm) for SL:DPC, indicated by black lines. (e) CSP comparison of PEBP1 encapsulated in 50:50 DLPC:DPC (gray) and 50:50 SL:DPC (pH 6.5; red), with significant shifts chosen by calculating a 20% trimmed mean plus 1σ (0.014 ppm) and 2σ (0.029 ppm) for SL:DPC (black lines). (f) CSP comparison of GPx4 in 50:50 DLPC:DPC (gray) and 70:30 SL:DPC (pH 6.0; red) nRMs against the aqueous state, with 20% trimmed mean plus 1σ (0.051 ppm) and 2σ (0.102 ppm) for SL:DPC shown in black lines. The (g) FABP4 (PDB: 3RZY), (h) PEBP1 (PDB: 1BD9), and (i) GPx4 (PDB: 2OBI), structural interaction maps show membrane interactions with the proteins. Unassigned residues are depicted in white, assigned residues and prolines in purple, residues with CPSs above the 20% trimmed mean plus 1σ are depicted in orange spheres, and residues above 20% trimmed mean plus 2σ are depicted in red spheres.

Optimized nRM conditions were used as a guide to encapsulate a series of PMPs within the SL:DPC nRMs; FABP4, PEBP1, and GPx4. The *W*_0_ was increased to 25 to better accommodate the PMPs which are larger than ubiquitin (8.6 kDa). All proteins were successfully encapsulated. Human FABP4 (14.6 kDa) was successfully encapsulated in a 70:30 SL:DPC mixture and verified by ^15^N‐HSQC and DLS, confirming a spherical RM with a solvodynamic diameter of 7.3 ± 1.9 nm with 69% of all residues able to be unambiguously mapped. We note that the human FABP4 protein used here contains bound endogenous *Escherichia coli* lipids which co‐purify with the protein, but nevertheless result in highly homogenous NMR spectra (Figure 4a). A 50:50 SL:DPC mixture also encapsulated FABP4 well, but where possible we ultimately selected nRM formulations with higher natural lipid percentages. PEBP1 (21.1 kDa) encapsulated well in 50:50 SL:DPC and resulted in high‐quality HSQC spectra (Figure 4b) with 73% of all residues unambiguously mapped, as did GPx4 (18.6 kDa) in the 70:30 mixture with 84% of all residues mapped unambiguously (Figure 4c). The GPx4 spectrum displays lower signal to noise and less signal intensity uniformity compared to the other spectra, which may be due to a variety of effects that are outlined in the discussion. DLS confirmed proper encapsulations with the solvodynamic diameters of 7.6 ± 1.9 nm and 7.3 ± 1.6 nm, respectively.

In order to verify the pH change upon encapsulation, we attempted to use 1D NMR with imidazole and phosphate internal standards. However, despite attempting with many solvent mixture conditions, we were unable to fully solubilize the lipid mixtures fully in order to construct a reproducible calibration curve, which is necessary for this approach (Marques et al., 2014; Smith & Luisi, 1980). Through these trials, even though full solubilization was never achieved, SL and PBL were observed to have a strong buffering capacity around pH 6.0 while the BHL did not have a strong buffering capacity. To determine the encapsulated pHs, we then moved to using the peak position of pH sensitive protein resonances as an internal standard for pH (Fuglestad, Marques, Jorge, et al., 2019; Marques et al., 2014). Great care must be taken to select appropriate resonances that are pH sensitive but are not sensitive to interaction with the nRM. We observed a pH shifting effect with FABP4 and PEBP1, which were both encapsulated in an aqueous phase containing buffer at pH 7.4 (FABP4) and 7.5 (PEBP1) for both SL and PBL nRMs. Resonance shifts consistent with a pH of 6.5 were observed when compared to aqueous pH titration spectra of PEBP1 using residue L58 (Figure S6). While there appears to be one assigned pH titratable residue in FABP4 (Figure S7), this is unreliable due to the location of the residue (K79) near the suspected membrane binding site, therefore the pH of encapsulated FABP4 was assumed to be comparable to the pH of encapsulated PEBP1 due to the similarity of buffers. GPx4 (aqueous pH 6.0) does not have a high‐quality pH indicator due to minimal shifting and resonances that do have pH shifting are sensitive to nRM interactions. Therefore, we cannot conclusively determine if there is a pH shift occurring when encapsulating GPx4 (Figure S8). However, since ubiquitin tends towards pH 6.0 and SL buffers in this pH range, it is reasonable to assume that encapsulated GPx4 is near pH 6.0 and used this aqueous pH for CSP comparisons. Additionally, since GPx4 has minimal pH dependent shifting, slight inaccuracies in pH will not greatly affect the mapping of the spectrum nor the CSPs.

After correcting for pH effects, FABP4 residues displaying resonance shifting upon SL:DPC encapsulation (Figure 4d) are consistent with a membrane binding region surrounding the helices that is speculated among some FABP family members (Storch & McDermott, 2009). This indicates that the membrane‐bound state is well captured within the nRM and is a significant result, since the membrane bound form of FABPs is difficult to capture due to its weak and transient nature (Lenz et al., 2023; Lu & Yang, 2021). Specifically, residues F4, K9, T29, G34, A36, K58, T60, T74, D76, and C117 show significant shifting greater than the 20% trimmed mean plus 1σ (0.04 ppm, Figure 4g). We find a number of resonances in FABP4 that correspond to residues in the α‐helices are not accounted for in the RM spectra, likely due to a very large chemical shift difference and difficulty mapping resonance shifts of this magnitude. A number of resonances appear in the central portion of the spectrum upon encapsulation, consistent with unfolding of the helical residues upon membrane interaction. This corresponds well with a proposed mechanism of fatty acid transfer by FABPs, that the central fatty acid‐binding cavity needs to open through localized protein unfolding to transfer fatty acids into or out of the membrane (Lenz et al., 2023; Xiao et al., 2020). Comparison of GPx4 and PEBP1 nRM ^15^N‐HSQC spectra to aqueous ^15^N‐HSQCs (Figure 4e, f) reveals interaction of both of these PMPs with the interior surface of the nRMs through their previously characterized membrane binding interfaces (Labrecque et al., 2022; Labrecque & Fuglestad, 2021). PEBP1 has several shifting resonances with CSPs greater than the 20% trimmed mean plus 1σ (0.015 ppm) including those corresponding to L41, Y81, W84‐H86, V107‐S109, R119, S142, G143, G147, and Y181. GPx4 shifting residues greater than 20% trimmed mean plus 1σ (0.051 ppm) are G46‐K48, V51, K105, D111, L116, I122, G126, K127, I129, G131, A133, I143, G147, K151, R152, G154, M156, and L166. Overall, the known PE ligand binding and membrane interaction region of PEBP1 is observed to interact with the interior surface of the nRM (Figure 4h) (Banfield et al., 1998; Labrecque et al., 2022), while the active site and membrane binding regions of GPx4 are likewise found to interact to the interior surface of the nRMs (Figure 4i) (Cozza et al., 2017; Labrecque & Fuglestad, 2021).

**FIGURE 5:**
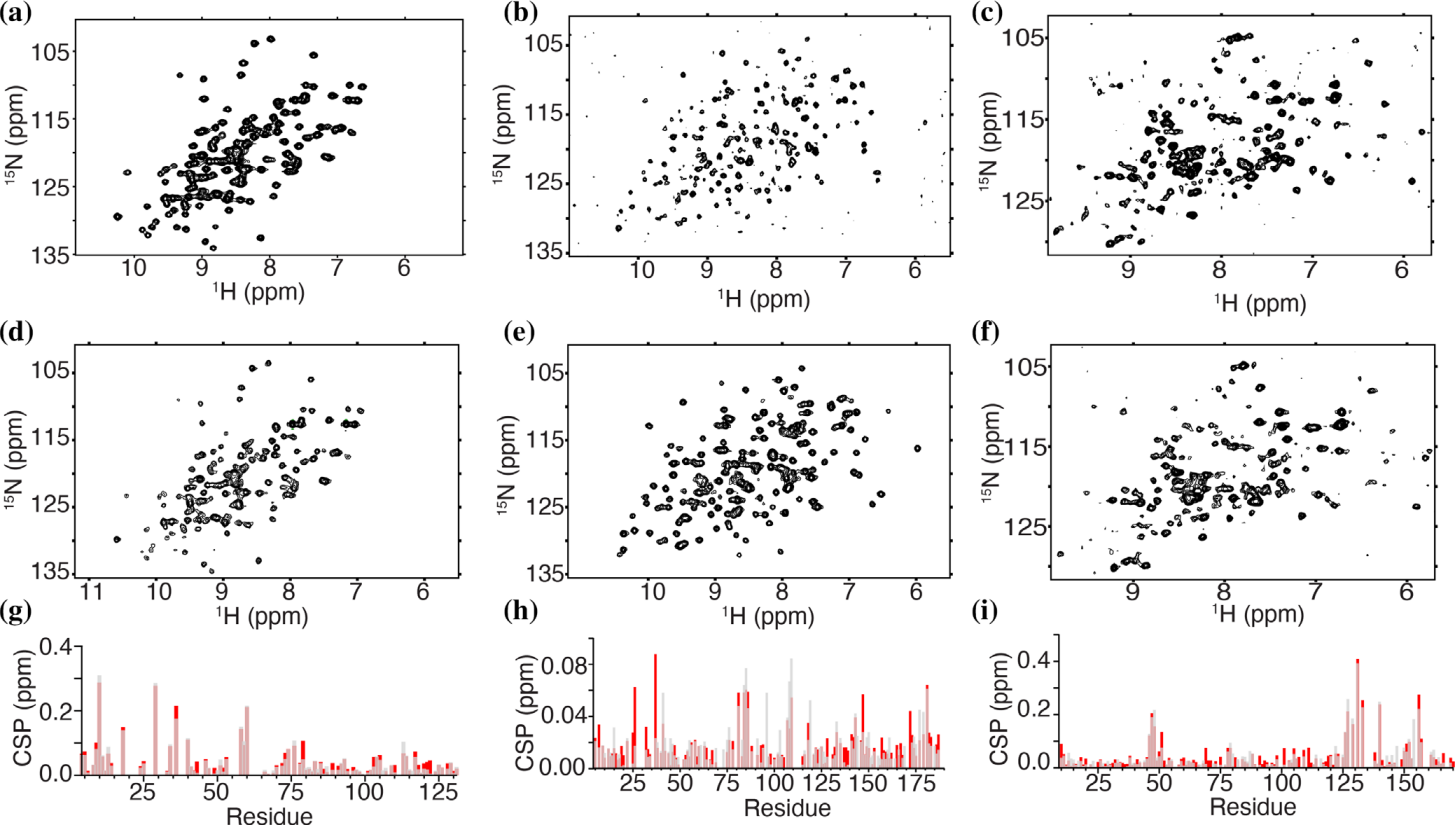
Encapsulation of FABP4, PEBP1, and GPx4 in optimized 50:50 porcine brain lipid (PBL):*n‐*dodecylphosphocholine (DPC) and bovine heart lipid (BHL):DPC native reverse micelles (nRMs), all with 75 mM total of lipid and DPC. (a) ^15^N‐HSQC of FABP4 encapsulated in 50:50 PBL:DPC nRM at a *W*_0_ of 25 with 450 mM hexanol. (b) ^15^N‐HSQC of PEBP1 encapsulated in PBL:DPC nRM at a *W*_0_ of 25 with 1.05 M hexanol. (c) ^15^N‐HSQC of GPx4 encapsulated in PBL:DPC nRM at a *W*_0_ of 20 with 500 mM hexanol. (d) ^15^N‐HSQC of FABP4 encapsulated in BHL:DPC nRM at a *W*_0_ of 25 with 950 mM hexanol. (e) ^15^N‐HSQC of PEBP1 encapsulated in BHL:DPC nRM at a *W*_0_ of 25 with 850 mM hexanol. (f) ^15^N‐HSQC of GPx4 encapsulated in BHL:DPC nRM at a *W*_0_ of 20 with 1.15 M hexanol. Chemical shift perturbation comparison of 50:50 BHL:DPC versus aqueous at original encapsulation pH for all (gray) and 50:50 PBL:DPC versus aqueous at pH 6.0 for all (red) of (g) FABP4, (h) PEBP1, and (i) GPx4 show interaction with the nRMs in the known membrane binding interfaces, with only minor differences between PBL:DPC and BHL:DPC nRM systems.

Following the same protocol, a mixture of PBL and DPC were used for encapsulation of PMPs, verified using ^15^N‐HSQC. These nRMs used 400–700 mM of hexanol for a 50:50 PBL:DPC encapsulation, as compared to needing at least 800 mM hexanol for a 50:50 SL:DPC mixture. The FABP4 best encapsulated at a *W*_0_ of 25 with a 50:50 PBL:DPC ratio and a hexanol concentration of 450 mM (Figure 5a). PEBP1 used a 50:50 PBL:DPC ratio for encapsulation at a *W*_0_ of 25 and a hexanol concentration of 1.05 mM (Figure 5b). PEBP1 was determined to have a pH decrease when encapsulated, going from the original pH of 7.5 to ~6.0 (Figure S6C). Since FABP4 does not have a reliable pH sensitive resonance (Figure S7), the pH change for PBL is based on the PEBP1 result and is assumed to shift from the original 7.4 to ~6.0. GPx4 best encapsulated with a 50:50 PBL:DPC ratio with a *W*_0_ = 20 and a hexanol concentration of 500 mM (Figure 5c). GPx4 was assumed to encapsulate at pH ~6.0 due to the PBL buffering capacity in this range and the results of the other protein pH shifts. CSPs for all three proteins in the PBL:DPC mixture versus in water matched well with the CSPs in the SL:DPC mixture, with small variations likely due to different lipid interactions (Figure 5g–i). We employed a similar process to encapsulate the PMPs within BHL:DPC nRMs. FABP4 and PEBP1 were successfully encapsulated in 50:50 BHL:DPC with a *W*_0_ = 25 (Figure 5d, e) and were determined to have a consistent pH when encapsulated in their original buffers, as determined by PEBP1 NMR pH titration (Figures S6D). The hexanol concentration for the encapsulation of FABP4 was 950 mM and for PEBP1 was 850 mM. Conditions for GPx4 encapsulation in BHL:DPC nRMs were extensively screened, but initial attempts were fruitless. Upon changing the pH of the aqueous buffer to 7.4, GPx4 was successfully encapsulated using 50:50 BHL:DPC, *W*_0_ = 20, and hexanol concentration of 1.15 M, though the ^15^N‐HSQC signal‐to‐noise was significantly lower, requiring a longer acquisition time (Figure 5f). Despite the ^15^N‐HSQC spectrum having lower signal‐to‐noise for GPx4 in BHL:DPC, the spectrum was of high enough quality to analyze CSPs. We observed very minor pH shifting in PEBP1 upon encapsulation in BHL nRMs (Figure S6D), consistent with the observed low buffering capacity, leading us to assume that BHL has little effect on pH for PEBP1, FABP4, or GPx4. The interior pH of the BHL:DPC mixtures is driven by the buffer in the aqueous phase, as opposed to the PBL:DPC and SL:DPC nRMs. CSPs for all proteins in BHL:DPC mixtures compared well to the other nRM formulations (Figure 5g–i).

## 3 DISCUSSION

Here we have demonstrated that PMPs can be encapsulated within nRMs constructed from naturally derived lipid extracts, which reflect the diversity of lipids found in cellular membranes. The characteristics of the nRMs developed well match with previously developed mmRMs, including size, shape, and dispersity of the nRMs. The nRMs also linearly increase in diameter when the *W*_0_ is increased, a common feature of RMs (De & Maitra, 1995; Fuglestad, Gupta, Wand, & Sharp, 2019; Mills et al., 2014; Palazzo et al., 2003). With many parameters that may be optimized, these systems offer a flexible method for many PMPs to be successfully encapsulated, highlighting its use as a tool for understanding structure, function, and dynamics of PMPs in a more biologically accurate model. The interactions of all three PMPs tested here match well with the known membrane interfaces, verifying that nRMs well‐mimic cellular membrane surfaces for study of proteins in their membrane embedded state.

Results highlighted here reveal the utility of using nRMs for studying membrane and lipid interactions with PMPs. nRMs capture the FABP4 membrane‐bound state and enable NMR studies. Direct observation of membrane‐bound FABPs is known to be challenging due to the transient nature of the protein‐membrane interaction and this marks an efficient method for study. While PEBP1 is efficiently housed within all of the tested nRM systems and interacts with the lipid surface, PEBP1 is known to interact with PE lipids, and PE is present at a larger percentage in the PBL and SL (>30%) compared to BHL (~13%) and DLPC:DPC mmRMs (none). PEBP1 is an example of a PMP with function suspected to be related to specific interacting lipids, making choice of lipid system important for selecting membrane models for study.

GPx4 is housed effectively in the nRM formulations tested, but generally results in lower signal‐to‐noise spectra than FABP4 and PEBP1. There are many possible reasons for this effect, such as a change in size or shape of the nRMs containing GPx4, μs‐ms conformational exchange induced in the protein upon embedment into the nRMs, a degree of dimerization brought on by nRM encapsulation, imperfection as models of biological membranes as a consequence of the single leaflet and extreme curvature in nRMs, among other potential explanations. Lower signal‐to‐noise for GPx4 compared to the other proteins highlights potential limitations of nRMs for certain PMPs and requires further investigation to determine the origin. However, CSP analysis is still possible and all nRM systems resulted in GPx4 in its membrane bound state, demonstrating that even for protein systems that do not have the best spectral qualities in nRMs, detailed surface maps of lipid and membrane interactions are easily obtained. In general, the PMPs encapsulated within nRMs have lower signal to noise compared to encapsulation within DLPC:DPC mmRMs. This may be a result of a larger RM particle size caused by the lipid alkyl chains being significantly longer than those in DPC or DLPC, slowing the overall tumbling and resulting in unfavorable relaxation effects. While these properties may limit the use of triple resonance NMR experiments in nRMs, this effect may be overcome by using lower viscosity alkanes such as ethane as the solvent (Peterson et al., 2005).

The nRMs demonstrated here add powerful new membrane models for high‐resolution studies of membrane‐interacting proteins. The formulations presented here are highly compatible with proteins for NMR studies, and proteins of varying sizes with molecular weights up to and likely beyond ~21 kDa are easily encapsulated and high‐quality NMR spectra are achieved. The formulation optimization approach outlined in this study may also be extended to a wide variety of lipid extracts, allowing flexibility in lipid content according to the biological context of the protein of interest. With these new tools, enhanced study of protein‐membrane interactions and other high‐resolution NMR studies such as structure, function, and dynamics, and other applications may be achieved with improved physiological relevance.

## 4 MATERIALS AND METHODS

### 4.1 Purification of soybean lecithin

To chelate and remove metal contaminants (Meneses & Glonek, 1988) from soy lecithin, 1 g of high purity SL (VWR International, USA) was solubilized in 15 mL of a chloroform: methanol (2:1) mixture in a glass vial. A 5 mL aliquot of aqueous 200 mM ethylenediaminetetraacetic acid (EDTA) pH 6.0 was added, and the mixture was stirred for 24 h at room temperature. The mixture was then separated via a separatory funnel, the organic layer was collected, and an additional 5 mL of aqueous 200 mM EDTA pH 6.0 was added after the chloroform: methanol mixture was increased to 15 mL. The separation step was repeated after mixing overnight and the organic layer was dried overnight at 37°C. Heavy metal purification was verified via sharp peaks in the ^1^H‐decoupled, ^31^P 1D NMR.

### 4.2 Formation of nRMs

To optimize the formation of nRMs, screens using 75 mM of purified lecithin and 75 mM total of various ratios (50:50 to 80:20) of an “empty” nRMs, or nRMs without protein, were formulated. Mass of lipid and DPC powders were added to a glass vial and vacuum dried overnight to ensure no additional water was introduced into the system. Pentane (Sigma‐Aldrich) was added to solubilize the surfactant system and 50 mM 1‐hexanol was added, which prevented a viscous gel‐like phase from forming upon adding water. Volumes of buffer corresponding to various water loading (*W*_0_) values were added to the nRM. *W*_0_ values ranged from 10 to 35 in increments of five. *W*_0_ is defined by the following formula:

$$W_{0}=\frac{\left[\text{water}\right]}{\left[\text{surfactant}\right]}$$

where the concentration of surfactant is the concentration of DPC plus the estimated total concentration of lipids within the lipid extract. 1‐hexanol was added into the system in 50 mM increments with vortexing in between additions until visual clarity was reached (Figure S1A). Phase diagrams using bromophenol blue in the aqueous phase were completed to gauge the necessary total 1‐hexanol addition for successful formation of best optimized formulations of nRMs (Figure S1B). The buffer system used for the empty nRM 20 mM Bis‐Tris pH 6.0, 100 mM NaCl, and 20 mM dithiothreitol (DTT) (GPx4 NMR buffer). Protein was added into the system by replacing the “empty” buffer system with the same volume of protein in its buffer. The correct volume was achieved via spin concentrating the sample.

### 4.3 Dynamic light scattering

Optimized surfactant systems with and without protein were verified for consistency and proper small, spherical formation via DLS. A water loading titration for all formations of each lipid (Soybean, bovine heart polar lipids, and porcine brain polar lipids [Avanti Polar Lipids]) composition were performed for *W*_0_ = 10, 20, and 30 of GPx4 NMR buffer. DLS was also performed on all protein encapsulations. Experiments were performed on a Malvern Zetasizer Nano‐S instrument. Viscosity and dielectric constant parameters were determined using prior literature of a binary system for hexanol with hexane (Franjo et al., 1995; Singh & Sinha, 1982). The nRM systems were loaded in a quartz cuvette and kept at room temperature. Each experiment was completed in triplicate with 12–18 scans per individual measurement.

### 4.4 Protein expression and production

Proteins were isotopically labeled with ^15^NH_4_Cl purchased from Sigma‐Aldrich. All proteins were grown from *E. coli* and induced with isopropyl‐ß‐D‐1‐thiogalactopyranoside (IPTG). Purification of GPx4, PEBP1, and FABP4 was performed using a Ni‐NTA affinity chromatography and purification of untagged ubiquitin occurred with cation exchange chromatography. N‐terminal polyhistidine tags were removed from GPx4, PEBP1, and FABP4. Final concentrations were determined via Bradford Assay. Full protein preparation details can be found in the Supporting Information S1.

### 4.5 NMR spectroscopy and analysis

All NMR samples were prepared with ^15^N‐isotopically labeled protein in its respective buffer. The GPx4 buffer consisted of 20 mM Bis‐Tris pH 6.0, 0.1 M NaCl, and 20 mM DTT. The PEBP1 buffer was composed of 25 mM Tris pH 7.5, 150 mM NaCl, 0.5 mM EDTA, and 5 mM DTT. The FABP4 buffer was 20 mM Tris pH 7.4, 100 mM NaCl, and 2 mM DTT. Ubiquitin NMR buffer was a solution containing 20 mM MES pH 5.5, 100 mM NaCl, and 1 mM DTT. For nRM samples, 10% d‐pentane (Sigma‐Aldrich) was used as the lock solvent. ^1^H‐^15^N‐HSQC experiments were collected on 600 and 700 MHz Bruker AVANCE III equipped with room temperature probes. ^1^H‐decoupled, ^31^P 1D NMR spectra were collected at 25°C on a 400 MHz Bruker AVANCE III instrument using a with either 10% d‐pentane or 10% d‐chloroform as the lock solvent. All experiments were collected at 25°C. All NMR data were processed with NMRPipe and analyzed using the NMRFAM‐Sparky distribution (Delaglio et al., 1995; Lee et al., 2015).

Assignments for aqueous ubiquitin were taken from those previously reported (BMRB 25972) (Surana & Das, 2016) and mapped to the spectrum of encapsulated ubiquitin as previously described (Labrecque et al., 2022). Human FABP4 assignments were made from previously published spectra of the delipidated form (Constantine et al., 1998), transferred to the aqueous spectrum of the lipidated form, which was then used to transfer assignments to the encapsulated spectra. PEBP1 and GPx4 assignments were transferred from published aqueous assignments (BMRB: 17382 and BMRB: 50955, respectively) (Labrecque & Fuglestad, 2021; Yi et al., 2011) to the RM‐bound forms as previously described (Labrecque et al., 2022).

Chemical shift perturbations were calculated using weighted chemical shifts in the following formula:

$$\mathrm{CSP}=\sqrt{{\left(\Delta^{1}H\right)}^{2}+{\left(\frac{\Delta^{15}N}{9.8655}\right)}^{2}}$$

Δ^1^H and Δ^15^N represent the changes in the ^1^H and ^15^N chemical shifts for each resonance.

## AUTHOR CONTRIBUTIONS

**Sara H. Walters:** Methodology; Visualization; Writing–original draft; Writing–review & editing; Investigation. **Abdul J. Castillo:** Writing–review & editing; Investigation; Data curation. **Angela M. Develin:** Data curation; Investigation; Writing–review & editing. **Courtney L. Labrecque:** Data curation; Investigation; Writing–review & editing. **Yun Qu:** Methodology; Writing–review & editing; Resources. **Brian Fuglestad:** Conceptualization; Funding acquisition; Data curation; Project administration; Supervision; Writing–review & editing; Writing–original draft.

## Supporting information


**Data S1.** Details for protein expression and purification are provided in the supplementary material.
**Table S1.** Approximate lipid compositions of the lipid extracts used in this study.
**Figure S1.** Images and phase diagrams for formation of empty nRMs based on SL:DPC nRM.
**Figure S2.** Effect of EDTA‐metal chelation and ^31^P NMR of lipid mixtures.
**Figure S3.** DLS size measurements of 70:30 SL:DPC.
**Figure S4.** Optimization of W_0_ with ubiquitin in 50:50 SL:DPC nRMs and pH effects.
**Figure S5.** NMR of encapsulated ubiquitin in BHL:DPC and PBL:DPC.
**Figure S6.** Determination of pH for PEBP1 in nRMs.
**Figure S7.** Attempt at pH determination for FABP4 in nRMs.
**Figure S8.** pH titration for GPx4 in aqueous solution.
