# Supplementary material for: Investigating protein‐membrane interactions using native reverse micelles constructed from naturally sourced lipids
Source: Protein Sci. 2023 Nov 1;32(11):e4786. doi: 10.1002/pro.4786 (PMC10578115; doi:10.1002/pro.4786)
Supplement: Supplementary file 1 — Data S1. Details for protein expression and purification are provided in the supplementary material. Table S1. Approximate lipid compositions of the lipid extracts used in this study. Figure S1. Images and phase diagrams for formation of empty nRMs based on SL:DPC nRM. Figure S2. Effect of EDTA‐metal chelation and 31P NMR of lipid mixtures. Figure S3. DLS size measurements of 70:30 SL:DPC. Figure S4. Optimization of W0 with ubiquitin in 50:50 SL:DPC nRMs and pH effects. Figure S5. NMR of encapsulated ubiquitin in BHL:DPC and PBL:DPC. Figure S6. Determination of pH for PEBP1 in nRMs. Figure S7. Attempt at pH determination for FABP4 in nRMs. Figure S8. pH titration for GPx4 in aqueous solution. [file PRO-32-e4786-s001.docx]

**Supporting Information for:**

**Investigating protein-membrane interactions using native reverse micelles constructed from naturally sourced lipids.**

Sara H. Walters^1^, Abdul J. Castillo^1^, Angela M. Develin^1^, Courtney L. Labrecque^1^, Yun Qu^1^, Brian Fuglestad^1,2^

^1^ Department of Chemistry, Virginia Commonwealth University, Richmond, Virginia 23284, United States.

^2^ Institute for Structural Biology, Drug Discovery and Development, Virginia Commonwealth University, Richmond, Virginia 23219, United States.

**Protein Expression**

The synthetic gene corresponding to residues 7-170 of the human cytosolic isoform of GPx4 with TEV cleavable poly-histidine tag was inserted into vector pNIC28-Bsa4. The selenocysteine at position 46 is mutated to glycine, yielding a catalytically inactive GPx4. The GPx4 encoding plasmid was a gift from Nicola-Burgess-Brown (Addgene plasmid #38797). The gene encoding human PEBP1 and the gene encoding human FABP4 were synthesized by Genescript and subcloned into pET-28a plasmids with a TEV cleavable poly-histidine tag. Human ubiquitin was inserted into the pET-3a vector. All plasmids were transformed into BL21 (DE3) *E. coli* which was grown on LB-agar plates with kanamycin or ampicillin. Glycerol stocks were made from a single colony from the growth and used to seed overnight starter cultures in M9 minimal media grown at 37°C. The overnight cultures were pelleted and used to grow cells in 1L M9 minimal media with 1g/L of ^15^NH_4_Cl as the source of nitrogen for ^15^N-labelling. Cells were grown in the M9 minimal media until an OD_600_ of 0.800 was measured. Induction was achieved with 1mM of isopropyl-ß-D-1-thiogalactopyranoside (IPTG) overnight at 30°C for GPx4 and ubiquitin or 18°C for PEBP1 and FABP4.

**Protein Purification**

GPx4 preparation followed a previously published protocol (Labrecque & Fuglestad, 2021). The cells were harvested via centrifugation and resuspended in lysis buffer [0.1 M Tris pH 7.4, 0.3 M NaCl, 0.5% v/v triton, 0.1 mg/mL lysozyme, protease inhibitor cocktail, and 5 mM dithiothreitol (DTT)] and then lysed via sonication. GPx4 was purified via a Ni-NTA affinity column with five column volumes of wash buffer [0.1 M Tris pH 8.0, 0.3 M NaCl, 50 mM imidazole, and 1 mM DTT] and three column volumes of elution buffer [0.1 M Tris pH 8.0, 0.3 M NaCl, 300 mM imidazole, and 1 mM DTT]. The His-tag was removed in overnight dialysis via an optimized TEV protease, produced in house using a plasmid encoding MBP-TEVcs(ENLYFQ/G)-His6-uTEV2Δ(220-242)-R5, a gift from Alice Ting (Addgene plasmid # 135456) (Sanchez & Ting, 2020). After proteolysis, the protein was repurified and dialyzed overnight in NMR buffer (20mM Bis-Tris pH 6.0, 0.1M NaCl, and 20mM DTT).

PEBP1 was prepared following previously published protocols (Labrecque et al., 2022; Yi, Peng, Guo, & Lin, 2011). The cells were lysed via sonication in buffer comprised of 50 mM NaH_2_PO_4_, 200 mM NaCl, 5 mM DTT and 1 mM AEBSF at pH 7.5. The protein was purified on a Ni-NTA affinity column and His-tag removed with TEV protease in overnight dialysis. The protein was exchanged in 25 mM Tris, 150 mM NaCl, 0.5 mM EDTA at pH 7.5. 5 mM DTT was added to protein samples before experimentation. Confirmation of purification was completed by SDS-PAGE.

Purification of untagged ubiquitin was prepared using a previously published protocol (Labrecque et al., 2022). Cells were lysed via sonication in buffer comprised of 100 mM Tris pH 7.4, 0.5% Triton v/v, 0.2 mg/mL lysozyme, protease inhibitor cocktail, and 1 mM DTT. Over an ice bath, 70% perchloric acid was added to the supernatant until a milky white color is held. The supernatant is centrifuged to remove aggregation and is dialyzed overnight in 50 mM sodium acetate pH 4.5 at 4°C. The following day, a cation exchange is performed on the Akta FPLC with two buffers (Buffer A: 50 mM sodium acetate pH 4.5, Buffer B: 50 mM sodium acetate pH 4.5 and 1 M NaCl) running a gradient of 0-40% of Buffer B. Fractions were collected and SDS-PAGE was run to separate out the monomeric ubiquitin. The fractions were then dialyzed overnight into NMR buffer [20 mM MES pH 5.5, 100 mM NaCl, and 1 mM DTT].

Cells with expressed FABP4 were lysed via sonication in the following buffer: 100 mM Tris pH 7.4, 500 mM NaCl, 0.5% v/v triton, 0.1 mg/ml lysozyme, protease inhibitor cocktail, and 2 mM DTT. The protein was purified over a Ni-NTA affinity column, and the His-tag was cleaved with optimized TEV protease (Sanchez & Ting, 2020) in an overnight dialysis in buffer containing 20 mM Tris pH 7.4, 100 mM NaCl, and 2 mM DTT. The next day, the protein was repurified using Ni-NTA resin and buffer exchanged into NMR buffer comprised of 20 mM Tris pH 7.4, 100 mM NaCl, and 2 mM DTT. Presence of bound, endogenous lipid from E. coli was confirmed using ^15^N-HSQC. The resulting spectrum was highly homogenous, indicating 100% of FABP4 was bound to lipid.

**Table S1.** Natural lipid extract compositions

| Lipid Headgroup | Soy Lecithin (%) | Porcine Brain Lipids (%) | Bovine Heart Lipids (%) |
| --- | --- | --- | --- |
| Phosphatidylcholine (PC) | 38.3 | 12.6 | 8.6 |
| Phosphatidylethanolamine (PE) | 32.0 | 33.1 | 13.6 |
| Phosphatidylinositol (PI) | 18.7 | 4.1 | 1.0 |
| Phosphatidylserine (PS) | 3.7^a^ | 18.5 | b |
| Phosphatidic acid (PA) | 2.8 | 0.8 | 0.6 |
| Lysophosphatidylethanolamine (LPE) | 2.2 | b | b |
| Phosphatidylglycerol (PG) | 2.2 | b | b |
| Cardiolipin (CL) | b | b | 1.7 |
| Cholesterol | c | ~20^d^ | ~8^d^ |
| Triacylglycerides | c | b | ~44^d^ |

Approximate compositions of lipid extracts used in this study. Remaining percentages are unknown and are anticipated to be composed of sterols, acylglycerides, and sphingolipids.

a. Lipid Headgroup ^31^P spectral peak is overlayed with LPC, therefore the PS and LPC together equal a percent composition of 3.72%.

b. Small percentages or none anticipated, not reported in composition analysis provided by Avanti Polar Lipids Inc. and not observed in ^31^P-spectra.

c. Small percentages or none anticipated, not observable in ^31^P-spectra.

d. Not reported, values estimated from published lipid tissue distribution analysis.(Das & Rouser, 1967; Lai et al., 2016)


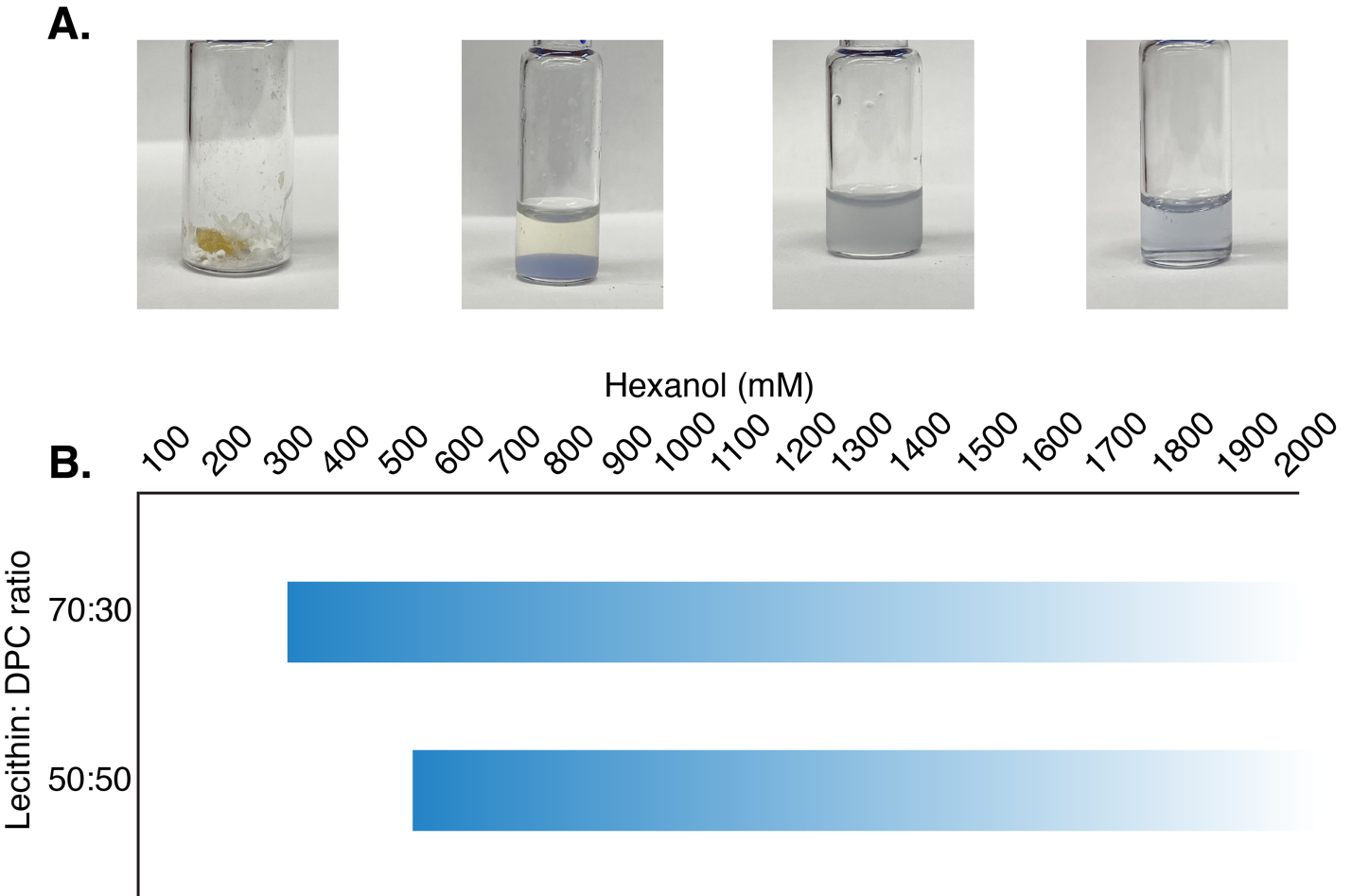


**Figure S1.** Images and Phase Diagrams for formation of empty nRMs based on SL:DPC nRM. **A.** Series of pictures depicting formation of nRMs. The first picture is the dry surfactants. The second picture is the after the addition of pentane and a W_o_ of 25 in buffer. The third picture is during the titration of hexanol and the fourth picture shows the visual clarity achieved when the encapsulation is complete. **B.** Phase diagram for 75 mM of SL:DPC nRM at W_o_ of 25. Titration was performed to understand hexanol tolerance of the nRMs. The blue bars indicate when the sample reached visual clarity. The titration was performed in increments of 50 mM (up to 1 M) then 100 mM increments to 2M. No phase separation was observed for either sample.


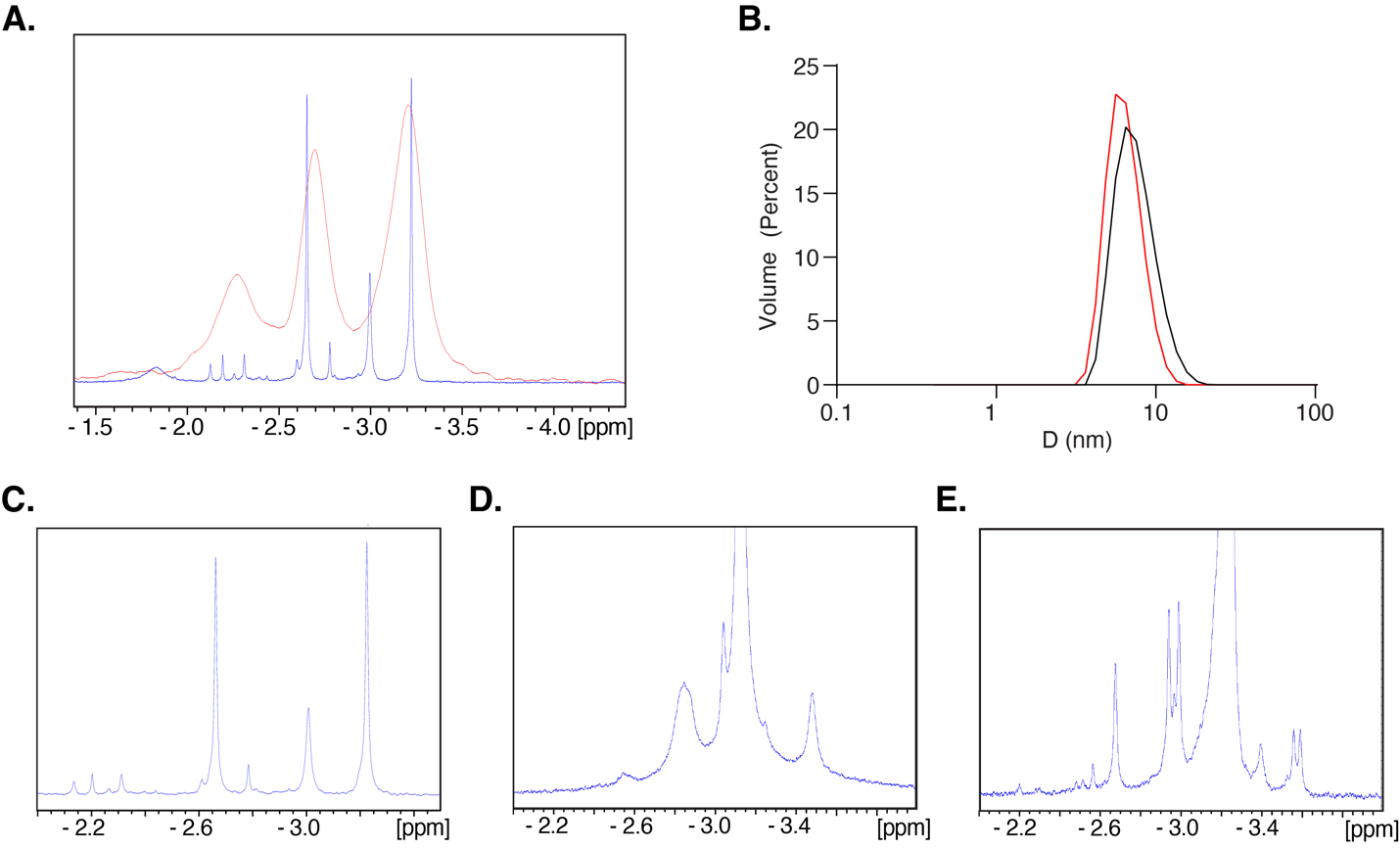


**Figure S2.** Effect of EDTA-metal chelation and ^31^P NMR of lipid mixtures. **A.** ^31^P NMR of SL before (red) and after (blue) heavy metal chelation, referenced to trimethyl phosphate. Broad peaks are an affect from paramagnetic metal contamination of the commercial soy lecethin, which is removed upon EDTA treatment. **B.** DLS of 50:50 SL:DPC nRM at W_o_ = 25 before and after heavy metal chelation: before chelation (black), 7.6 ± 2.3 nm and after chelation (red), 6.5 ± 1.7. Error represents the standard deviation of the size distribution determined by DLS. **C.** ^31^P NMR of 100% soy lectithin referenced to internal standard of trimethyl phosphate, used for quantification of lipid content in the EDTA-processed soy lecethin. **D.** ^31^P NMR of 50:50 porcine brain lipids:DPC empty nRM (Avanti Polar Lipids) referenced to trimethyl phosphate. **E.** ^31^P NMR of 50:50 bovine heart lipids:DPC empty nRM (Avanti Polar Lipids) referenced to trimethyl phosphate.

**
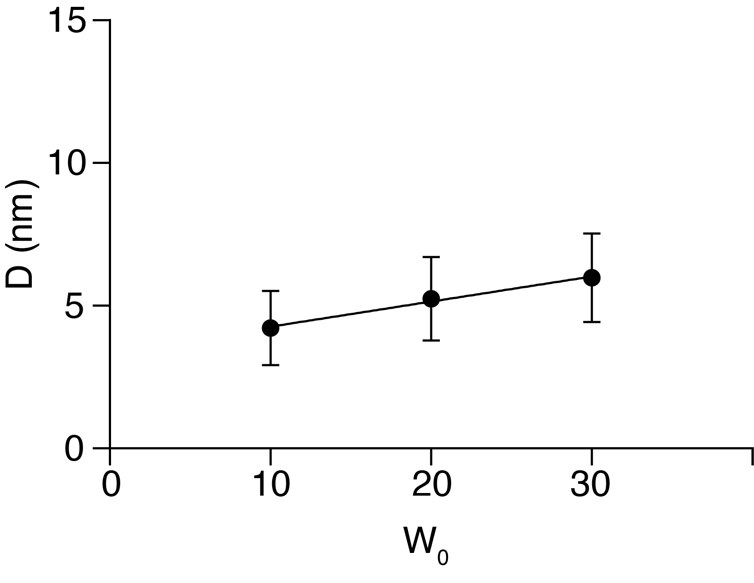
**

**Figure S3.** DLS size measurements of empty nRMs formulated from 75 mM mixture of 70:30 SL:DPC at Wo = 10 (4.2 ± 1.3 nm), 20 (5.2 ± 1.5 nm), and 30 (6.0 ± 1.6 nm). Error bars represent the standard deviation of the size distribution determined by DLS.

**
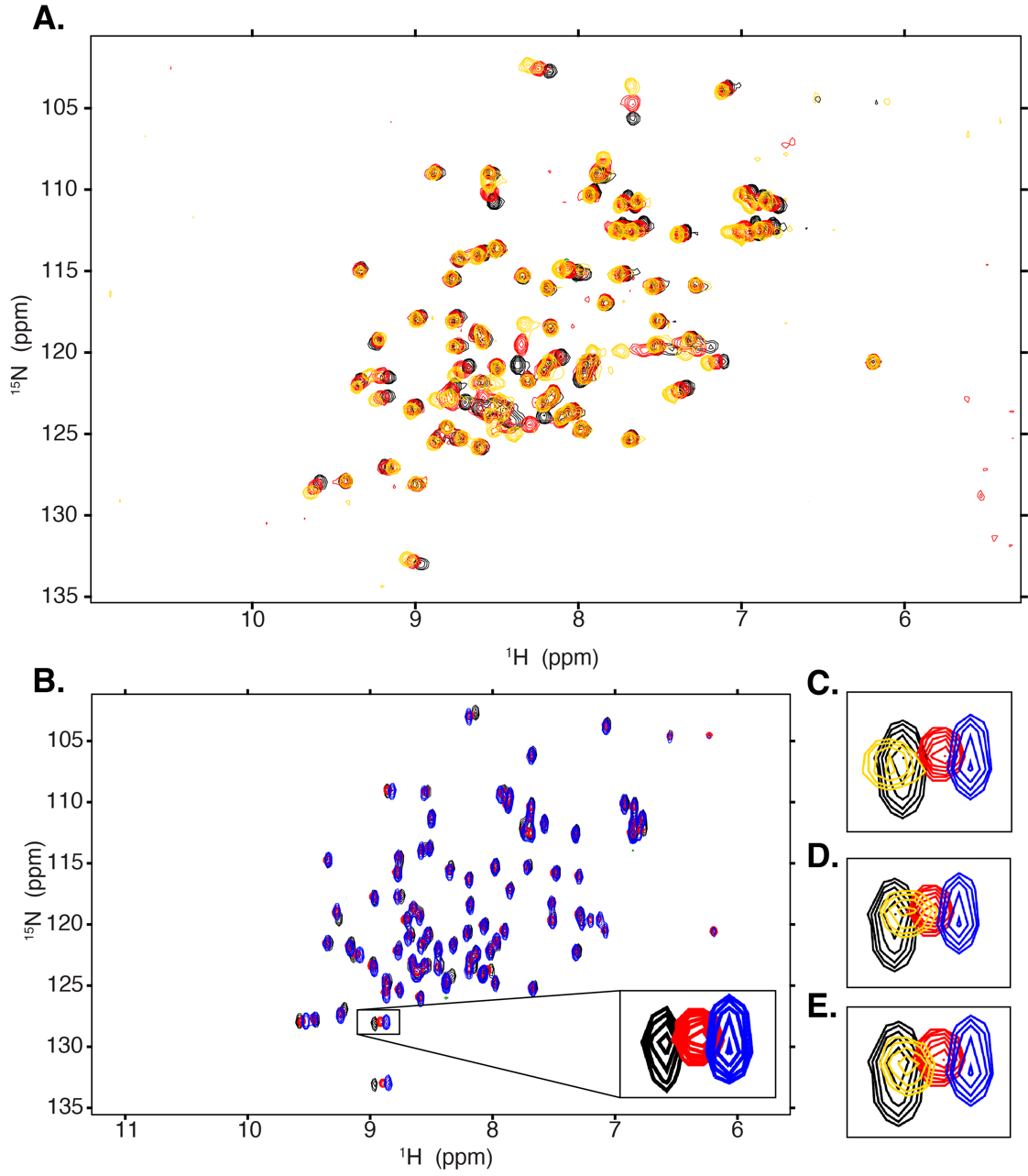
**

**Figure S4.** nRM optimization with encapsulated Ubiquitin and pH determination. **A.** ^15^N-HSQC of ubiquitin in 75 mM 50:50 SL:DPC nRM formulated with 500 mM hexanol and various water loading values; W_o_ of 10 (yellow), 15 (red), and 20 (black). **B**. Aqueous pH titration of ubiquitin (black = 6.0, red = 5.5, blue = 5.0). Inset is of K6, a pH sensitive resonance that does not have observed interactions with the nRMs **C.** Insert overlaying a SL:DPC encapsulated ubiquitin peak (yellow) over the pH titration series. **D**. Insert overlaying a PBL:DPC encapsulated ubiquitin peak (yellow) over the pH titration series. **E**. Insert overlaying a BHL:DPC encapsulated ubiquitin peak (yellow) over the pH titration series.


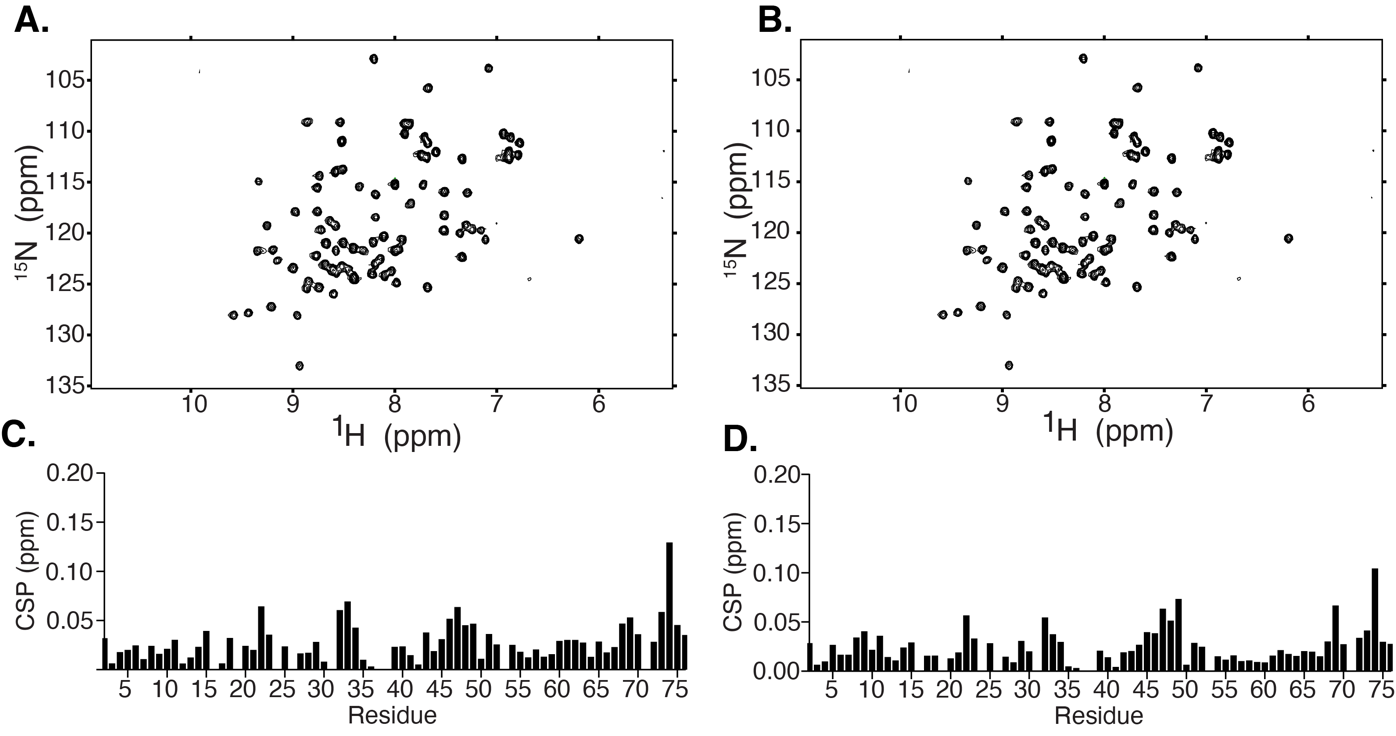


**Figure S5.** Encapsulated ubiquitin in BHL:DPC and PBL:DPC. **A.** ^15^N-HSQC of ubiquitin in a 75 mM 50:50 PBL:DPC nRM at a W_o_ of 20 with 1.2 M hexanol. **B.** ^15^N-HSQC of ubiquitin in a 75 mM 50:50 BHL:DPC nRM at a W_o_ of 20 with 600 mM hexanol. CSPs of ubiquitin encapsulated in **C.** 50:50 PBL:DPC and **D.** 50:50 BHL:DPC RMs compared against the pH = 6.0 aqueous protein, displaying anticipated shifting of surface residues due to weak anionic interactions.


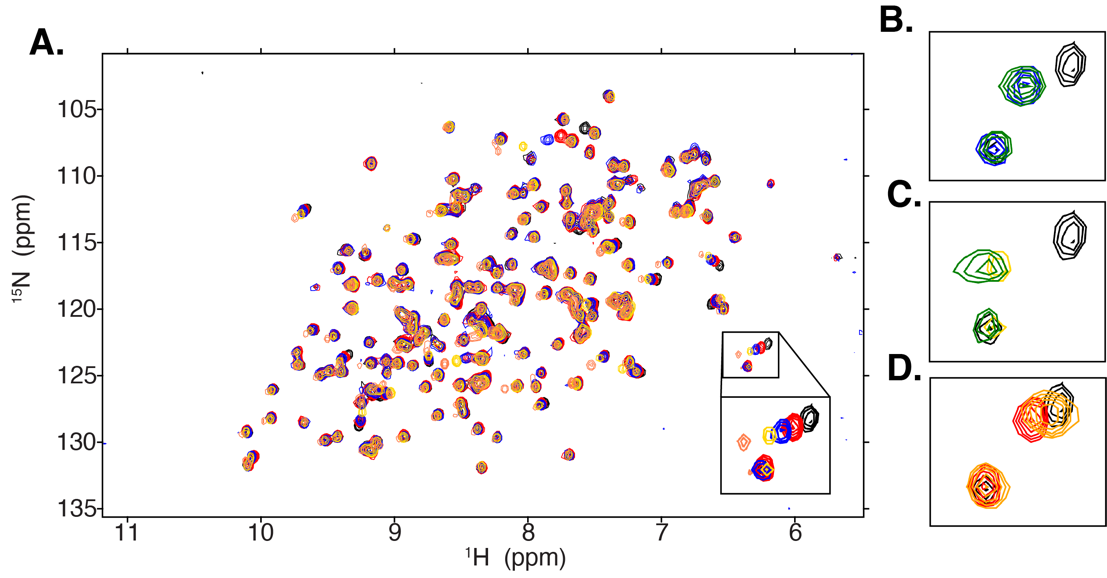


**Figure S6.** Determination of pH for PEBP1 in nRMs based on aqueous ^15^N-HSQC. **A.** ^15^N-HQSC aqueous pH titration (black = 7.5, red = 7.0, blue = 6.5, yellow = 6.0, coral = 5.0) including insert showing pH shifting peak (L58) that was used for pH determination. **B.** SL:DPC nRM pH shifting resonance L58 (green) overlaying with pH ~6.5. **C.** PBL:DPC nRM pH shifting resonance L58 (green) overlaying with pH ~6.0. **D.** BHL:DPC nRM pH shifting resonance L58 (orange) overlaying with pH ~7.5.


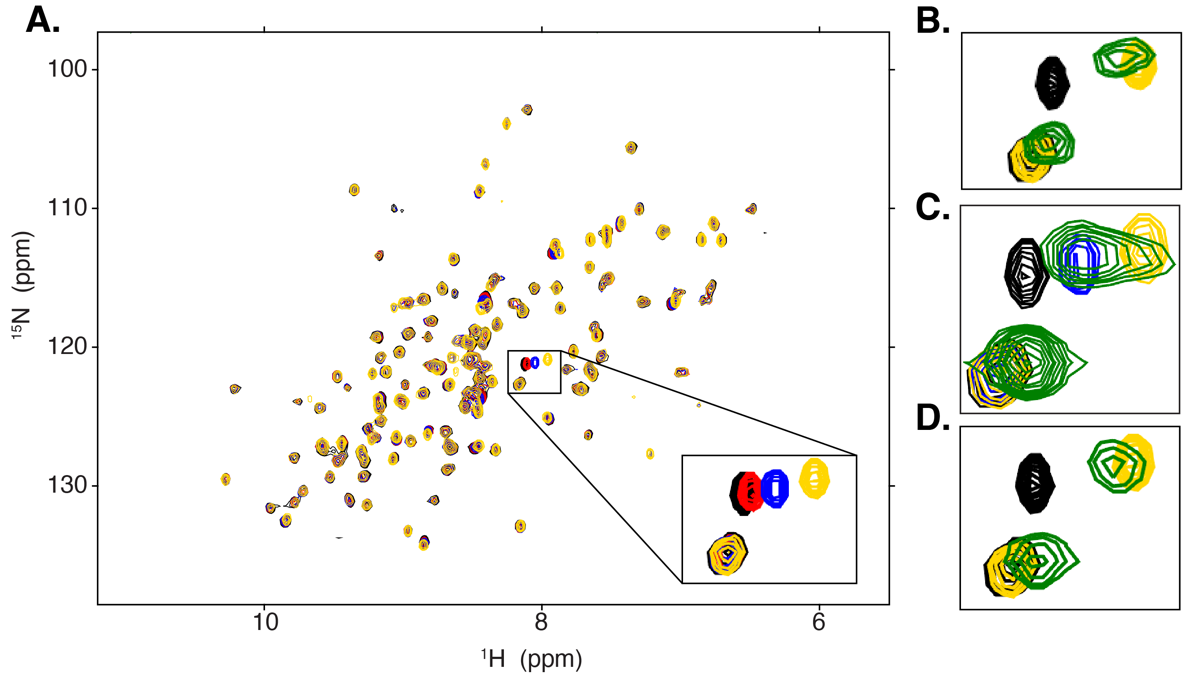


**Figure S7.** Attempt at pH determination for FABP4 in nRMs based on aqueous ^15^N-HSQC. The only resonance with a large degree of pH sensitive shifting, K79, is in the region that interacts with the nRM and thus could not be used for pH calibration. **A.**^15^N-HQSC aqueous pH titration (black = 7.5, red = 7.0, blue = 6.5, yellow = 6.0) including insert showing pH shifting peak corresponding to K79 **B.** SL nRM pH shifting residue (green) overlaying with pH 6.0. **C.** PBL nRM shifting residue (green) overlaying with pH 6.5. **D.** BHL nRM shifting residue (green) overlaying with pH 6.0. This serves as an example of an unreliable protein resonance shift for pH calibration.


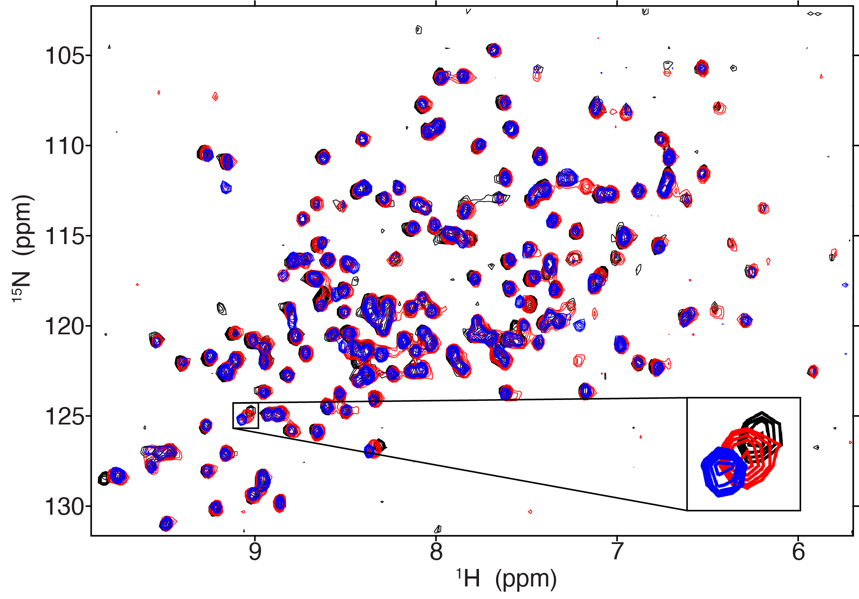


**Figure S8.** pH titration for GPx4 in aqueous solution using ^15^N-HSQC. ^15^N-HQSC pH titration (black = 7.0, red = 6.5, blue = 5.5) with inset zoomed on residue M26. Only minor peak shifting is observed as a function of pH in GPx4 and the shifting peaks are unreliable as indicators due to some sensitivity to nRM interactions.

**References**

Das, M. L., & Rouser, G. (1967). Lipid composition of beef heart ventricle. *Lipids*, *2*(1), 1–4.

Labrecque, C. L., & Fuglestad, B. (2021). Electrostatic Drivers of GPx4 Interactions with Membrane, Lipids, and DNA. *Biochemistry*, *60*(37), 2761–2772. doi: 10.1021/acs.biochem.1c00492

Labrecque, C. L., Nolan, A. L., Develin, A. M., Castillo, A. J., Offenbacher, A. R., & Fuglestad, B. (2022). Membrane-Mimicking Reverse Micelles for High-Resolution Interfacial Study of Proteins and Membranes. *Langmuir*, *38*(12), 3676–3686.

Lai, Y., Choi, U. B., Zhang, Y., Zhao, M., Pfuetzner, R. A., Wang, A. L., … Brunger, A. T. (2016). N-terminal domain of complexin independently activates calcium-triggered fusion. *Proceedings of the National Academy of Sciences*, *113*(32), E4698–E4707.

Sanchez, M. I., & Ting, A. Y. (2020). Directed evolution improves the catalytic efficiency of TEV protease. *Nature Methods*, *17*(2), 167–174.

Yi, C., Peng, Y., Guo, C., & Lin, D. (2011). 1H, 13C, 15N backbone and side-chain resonance assignments of the human Raf-1 kinase inhibitor protein. *Biomolecular NMR Assignments*, *5*(1), 63–66. doi: 10.1007/s12104-010-9268-z
